# Multinational outbreak of travel-related *Salmonella* Chester infections in Europe, summers 2014 and 2015

**DOI:** 10.2807/1560-7917.ES.2017.22.7.30463

**Published:** 2017-02-16

**Authors:** Laure Fonteneau, Nathalie Jourdan Da Silva, Laetitia Fabre, Philip Ashton, Mia Torpdahl, Luise Müller, Brahim Bouchrif, Abdellah El Boulani, Eleni Valkanou, Wesley Mattheus, Ingrid Friesema, Silvia Herrera Leon, Carmen Varela Martínez, Joël Mossong, Ettore Severi, Kathie Grant, François-Xavier Weill, Céline M Gossner, Sophie Bertrand, Tim Dallman, Simon Le Hello

**Affiliations:** 1 Santé publique France, the French national public health agency, Saint-Maurice, France; 2 European Programme for Intervention Epidemiology Training (EPIET), European Centre for Disease Prevention and Control (ECDC), Stockholm, Sweden; 3 Institut Pasteur, French National Reference Center for E. coli, Shigella and Salmonella, Paris, France; 4 Public Health England, Gastrointestinal Bacterial Reference Unit, London, England; 5 Statens Serum institut (SSI), Denmark; 6 Institut Pasteur du Maroc, Sécurité alimentaire et environnement, Casablanca, Morocco; 7 University Ibn Zohr, LBVRN, Agadir, Morocco; 8 NRL Salmonella & AMR, Veterinary Laboratory of Chalkida, Greece; 9 NRL Salmonella, Institute of Public Health, Belgium; 10 Netherlands National Institute for Public Health and the Environment (RIVM), the Netherlands; 11 National Center for Microbiology. Instituto de Salud Carlos III, Spain.; 12 Instituto de Salud Carlos III, CIBER Epidemiología y Salud Pública (CIBERESP), Spain; 13 Laboratoire National de Santé, Luxembourg; 14 European Centre for Disease Prevention and Control, Stockholm, Sweden; 15 School of Public Health and Primary Care (CAPHRI), Maastricht University Medical Center (MUMC+), Maastricht, the Netherlands

**Keywords:** *Salmonella*, outbreak, serotype Chester, Morocco, case-case study, WGS

## Abstract

Between 2014 and 2015, the European Centre for Disease Prevention and Control was informed of an increase in numbers of *Salmonellaenterica* serotype Chester cases with travel to Morocco occurring in six European countries. Epidemiological and microbiological investigations were conducted. In addition to gathering information on the characteristics of cases from the different countries in 2014, the epidemiological investigation comprised a matched case–case study involving French patients with salmonellosis who travelled to Morocco that year. A univariate conditional logistic regression was performed to quantify associations. The microbiological study included a whole genome sequencing (WGS) analysis of clinical and non-human isolates of *S*. Chester of varied place and year of isolation. A total of 162 cases, mostly from France, followed by Belgium, the Netherlands, Spain, Denmark and Sweden were reported, including 86 (53%) women. The median age per country ranged from 3 to 38 years. Cases of *S*. Chester were more likely to have eaten in a restaurant and visited the coast of Morocco. The results of WGS showed five multilocus sequence types (ST), with 96 of 153 isolates analysed clustering into a tight group that corresponded to a novel ST, ST1954. Of these 96 isolates, 46 (48%) were derived from food or patients returning from Morocco and carried two types of plasmids containing either *qnrS1* or *qnrB19* genes. This European-wide outbreak associated with travel to Morocco was likely a multi-source outbreak with several food vehicles contaminated by multidrug-resistant *S*. Chester strains.

## Introduction

Non-typhoidal *Salmonella* infections are the most common cause of reported food-borne outbreaks in the European Union (EU) [1,2]. These infections mostly cause mild disease (gastroenteritis), however life-threatening infections (e.g. bacteraemia) may occur, particularly in cases involving patients at the extremes of age or who are immunocompromised. Due to the large animal reservoir, including farm animals, pets and wild animals, *Salmonella* is mainly transmitted by consumption of contaminated food and to a lesser extent by contaminated environments, contact with animals, or person-to-person [3]. The mean incubation period is between 1 and 3 days. More than 2,500 serotypes of the genus *Salmonella* have been described so far [4]. 

Of these, serotype Chester is not commonly identified through human surveillance. Between 2009 and 2013, EU and European Economic Area (EU/EEA) countries reported through The European Surveillance System (TESSy) a mean of 91 *S.* Chester cases per year, which accounts for only 0.1% of all annual salmonellosis cases notified in the EU/EEA [5]. Outbreaks associated with *S.* Chester have been reported: in Australia, associated with sea turtle meat in 1998 and with tap water in 2005; in the United States, associated with cantaloupe in 1990 and with frozen meals (cheesy chicken and rice) in 2010; in Japan associated with cuttlefish chips in 1999 and in Canada, associated with headcheese in 2010 [6-11]. *S.* Chester was also the second most common serotype in poultry, in 2010, in Burkina Faso [12]. From 2005 to 2015, according to the Rapid Alert System for Food and Feed (RASFF, http://ec.europa.eu/food/safety/rasff/index_en.htm) database, a EU tool to share information when cross-border risks to public health are detected in the food chain, *S.* Chester was found in kangaroo meat (twice in 2007 and 2011 respectively), peppermint (once in 2005), dog chew (once in 2005) and fishmeal (six times in 2014) [13].

In France, the human *Salmonella* surveillance system is based on a voluntary network of laboratories that send or report their *Salmonella* isolates to the French National Reference Center for *Escherichia coli*, *Shigella* and *Salmonella* (NRC) [14,15]. Travel information is collected from laboratory surveillance forms (completed for ca 30% of the patients in 2014). In addition, food-borne outbreaks of salmonellosis (at least two cases clustered in time and place) are subject to mandatory notification to the French Institute for Public Health Surveillance (Santé publique France; SpF).

In September 2014, the French NRC notified SpF of an increase in numbers of *S.* Chester isolates, with 31 isolates received between August and September 2014, slightly more than twice the number observed for the same period in 2013 (n = 14). Most cases had travelled to Morocco within two weeks prior to their symptom onset. During the same period, Belgium had initiated a similar notification to the European Epidemic Intelligence Information System (EPIS) of the European Centre for Disease Prevention and Control (ECDC) with 18 *S.* Chester cases. The Netherlands, Spain and Denmark reported clusters of, respectively eight, six, and four cases, and Sweden reported one case [1]. In September 2014, a European investigation was launched in order to identify the vehicle(s)/source(s) of infection and implement control measures. France, the country with the highest number of cases, coordinated this investigation with the support of the ECDC.

In this article, we describe the epidemiological and microbiological investigations of the outbreak and report and discuss their outcome. 

## Methods

We carried out both epidemiological and microbiological investigations. The epidemiological investigation only included cases with symptom onset in 2014 while the microbiological investigation considered cases with onset occurring over a larger time frame as further described.

### Epidemiological investigation

#### Case definition

We defined a case as a symptomatic resident of the EU/EEA with laboratory-confirmed *S.* Chester infection and with symptom onset (or date of strain isolation in case of unavailable onset date) between week 17 (April) and week 41 (October) of 2014.

#### Descriptive analysis and exploratory investigation

We described cases in terms of age, sex and travel history to Morocco.

In France, we interviewed the most recently infected cases (with isolates obtained from week 33 of 2014) using a trawling questionnaire. This gathered information on demographics, clinical details, travel within the seven previous days with a focus on Morocco, contact with symptomatic persons, attended events, visited places and a detailed food history for the week before symptom onset. If the case was a child aged less than 15 years, we interviewed one of his/her parents.

The Dutch and Belgian Institutes used the same questionnaire to interview their cases. The Danish and Spanish cases were interviewed using a different questionnaire focusing on travel destination and whereabouts during the travel. The Swedish case was not interviewed on exposures.

#### Case–case study in France

In France, SpF carried out a matched case–case study to test the hypotheses raised by the exploratory investigation. We interviewed cases, with symptom onset between week 31 and week 40, who were not included in the exploratory investigation. We selected controls, among non-typhoidal *Salmonella* cases, who were diagnosed with an infection by other serotypes than Chester, who reported travel history to Morocco in the week before symptoms and whose symptoms started between week 27 and week 40 of 2014. We selected two controls for each case. After excluding cases who did not travel to Morocco before being symptomatic, we performed a crude analysis and three matched analyses. We separately matched cases with controls according to their age group (< 1, 1–5, 6–15, 16–40, > 40 years of age), according to the week of their symptom onset (plus or minus two weeks) and according to both their age group and week of symptom onset.

We performed a univariate conditional logistic regression to quantify associations. We calculated matched odds ratios (mORs) and their 95% confidence interval (CI). We used Stata v12.1 (Stata Corporation, Texas, US) for analysis.

### Microbiological investigations conducted at the European Union level

#### *Salmonella* Chester isolates

A microbiological investigation was conducted at the EU level, whereby countries were also asked whether they could participate in a whole genome sequencing (WGS) study. Five EU countries consisting of England and Wales (which conduct WGS routinely), France, Denmark, Belgium and Luxemburg, took part, making, overall, a total of 153 *S.* Chester isolates available for the investigation. One hundred and forty seven human isolates were selected so as to reflect a significant diversity in terms of year of isolation, geographical area of acquisition and potential link with the present multinational outbreak. Six non-human isolates from 2014 and 2015 were also added. Of the 147 human isolates, 82 were isolated in England and Wales between 2012 and 2015, 45 in France between 2011 and 2015 (including 26 isolates obtained during the epidemiological investigation), nine in Denmark in 2014 and 2015 (including 6 from the epidemiological investigation), six in Belgium in 2014 (all were from the epidemiological investigation) and three in Luxemburg between 2013 and 2014. The two remaining isolates were the reference strains (17K and ATCC 11997). 17K represents the historical reference strain first isolated from humans during a food poisoning outbreak in the hospital of Chester, United Kingdom (UK) in 1937 [16] and the ATCC 11997 is a reference strain from the Centers for Disease Control and Prevention of the United States (US CDC) [17]. Among these 145 patients, 71 (49%) reported international travel two weeks before illness onset (mainly in Morocco, n = 42 and West African countries, n = 10), 34 reported no travel and for the 40 remaining patients, this information was unknown. Of the six non-human isolates, three were collected from food (two from chicken sausages in Casablanca, Morocco and one from poultry in Belgium), one from decanted water in a treatment plant in Agadir, Morocco, one from turkey meat isolated in France but imported from Spain and the remaining isolate was isolated from fishmeal. Contrary to the other isolates obtained from random controls, this latter strain was sent upon request by the Greek authorities to the NRC in 2015 after a notification through RASFF about border rejection of fishmeal from Morocco in October 2014.

For all isolates sent to the French NRC (including the six human isolates from Belgium, the 45 human isolates from France and the six non-human isolates), the serotype was confirmed by agglutination tests with antisera (Bio-Rad, Marnes-la-Coquette, France) according to the White-Kauffmann-Le Minor scheme [4]. For the 82 English and Welsh, the nine Danish and the three Luxemburgish isolates, the serotype was determined from genome sequences, which were shared.

#### Antimicrobial susceptibility testing

A total of 105 *S.* Chester isolates were selected for antimicrobial susceptibility testing (AST). These consisted of 35 of the 82 isolates from England and Wales, and all human isolates from France (n=45), Denmark (n=9), Belgium (n=6), Luxemburg (n=3) as well as the six hon-human isolates and the 17K reference strain. AST was carried out by the disk diffusion method, with a panel of 32 antimicrobial drugs (Bio-Rad) as previously described [18]. The minimal inhibitory concentration (MIC) of nalidixic acid, ciprofloxacin, azithromycin and colistin were determined by using Etests (BioMérieux, Marcy l’Etoile, France) and interpreted according to the European Committee on Antimicrobial Susceptibility Testing (EUCAST) clinical guidelines [19].

#### Pulsed-field gel electrophoresis 

PulseNet standard pulsed-field gel electrophoresis (PFGE) of *Xba*I-digested chromosomal DNA was performed on a subset of 36 isolates. PFGE profiles were compared using Bionumerics software, v6.6 (Applied Maths, Sint Martens Latem, Belgium) and by the molecular typing clusters detection tools of ECDC.

#### Whole genome sequencing

For English and Welsh, French, Danish, Belgian and Luxemburgish *S.* Chester strains, genomic DNA was extracted and purified using different kits (Wizard of Promega or QiaAmp of Qiagen) and DNA samples were processed according to Illumina systems (MiSeq, NextSeq or HiSeq) generating 150 bp paired-end reads. Sequences were transferred to NRC for compiled analysis. Reads were trimmed and filtered using AlienTrimmer [20] with a quality Phred score threshold of 28 on a minimum length of 30 nt. De novo assembly was performed with SPAdes assembler version v2.5.1 [21]. Assembled sequences were analysed using web-tools available from the Center for Genomic Epidemiology (CGE) website (http://www.genomicepidemiology.org/) to obtain the multilocus sequence typing (MLST) type, to detect resistance genes (ResFinder) and to detect and type plasmids (PlasmidFinder and pMLST). New MLST types were confirmed by Sanger sequencing according to the MLST database website (http://mlst.warwick.ac.uk/mlst/).

As there is no complete *S.* Chester reference genome in public databases, core-genome multi-alignment of assembled genomes was done using harvest v1.0.1 f parsnp function [22] against the 17K reference strain or the ATCC 11997 *S.* Chester assembly [16,17]. The software uses FastTree2 to infer an approximately maximum-likelihood phylogenetic tree based on the analysis of 11,879 chromosomal single-nt polymorphisms (SNPs) from the 153 short read sequences of *S.* Chester [23]. The final tree was visualised in FigTree version 1.4.2 (http://tree.bio.ed.ac.uk/software/figtree/).

#### Phylogenetic analysis of strains with sequence type 1954

A sequence type (ST)1954 specific *S.* Chester phylogeny was constructed. ST1954 short-read sequences were mapped to the SPAdes v2.5.1 [21] de novo assembly of isolate 60056_H14424061601–2 using BWA-MEM [24]. SNPs were identified using GATK2 [25] in unified genotyper mode. Genome positions that had a high quality SNP (> 90% consensus, minimum depth 10x, GQ ≥ 30) in at least one isolate were extracted. Pseudosequences of polymorphic positions were used to create maximum likelihood trees using RAxML [26] and pairwise SNP distances between each pseudosequence calculated. Hierarchical single linkage clustering was performed on the pairwise SNP difference between all isolates at various distance thresholds (Δ250, Δ100, Δ50, Δ25, Δ10, Δ5, Δ0). The result of the clustering is a SNP address that can be used to describe the population structure based on clonal groups [27].

#### Sequence accession codes

FASTQ sequences were deposited in the National Center for Biotechnology Information (NCBI) Short Read Archive under the BioProject PRJNA248792.

## Results

### Epidemiological investigations

#### Descriptive epidemiology

**Figure 1:**
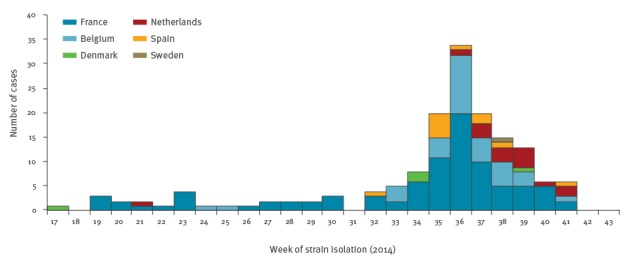
Distribution of *Salmonella* Chester cases by respective isolate week and country of residence, European Union, 2014 (n = 162)

Between week 17 and 41 of 2014, six EU countries (France, Belgium, the Netherlands, Spain, Denmark and Sweden) reported 162 cases through EPIS. The number of reported cases peaked on the first week of September (week 36, 2014) (Figure 1).

Of the 162 EU cases, about half of the cases (86; 53%) were women and the median age ranged from 3 to 38 years according to the country of notification. We obtained the travel history for 55 cases and 45 (82%) had recently travelled to Morocco (Table 1).

**Table 1:** Characteristics of *Salmonella* Chester cases, European Union, 2014 (n=162)

**Characteristics**	**France (n = 90)**	**Belgium (n = 35)**	**Netherlands (n = 15)**	**Spain (n = 11)**	**Denmark (n = 10)**	**Sweden (n = 1)**	**All** **(n = 162)**
**Proportion of women n (%)**	51 (57)	15 (43)	10 (67)	6 (55)	4 (40)	0	86 (53)
**Median age (years)**	3	14.5	5	6	38	NA	NA
**Number of cases with known travel information**	20	8	12	5	9	1	55
**Number of cases that travelled to Morocco**	17	8	10	5	4	1	45

NA: not applicable.

#### Exploratory investigation

In France, 16 cases were interviewed (8 females, 8 males) with a median age of 2 years (range: 1–32 years). Four cases aged between 1 and 3 years had been hospitalised (median length of hospitalisation: 5.5 days). Fifteen of the 16 cases had travelled to Morocco before symptom onset, staying there between two and six weeks. In the following analysis, we describe these 15 cases. The majority (10 of 15) arrived in Morocco by car and boat through the ports of Tanger (n = 7), Nador (n = 1) or Ceuta (n = 2), five cases travelled by plane landing in different airports in Morocco. The period between the date of arrival in Morocco and the symptom onset was always longer than seven days (median: 20; range: 8–48). We did not identify any common place (city, hotel, restaurant, supermarket) or activity shared by all cases. The food exposures most frequently mentioned were ice cream (14 of 15 cases; 14/15), grapes (10/11), chicken (13/15), pasteurised milk (13/15) and spreadable cheese (13/15). Shrimps were mentioned by five of the 15 cases. Eleven of the 15 the cases mentioned eating in a restaurant located in different cities.

In the Netherlands, 10 cases were interviewed, in Belgium, seven, in Spain, eight and in Denmark, nine. The food exposures most frequently mentioned were chicken (22 cases of 25 for which this information was available), grapes (17/20) and pasteurised milk (21/25).

#### Case–case study in France

Eighteen cases and 26 control-cases were interviewed, four of the 18 cases were excluded because they did not travel to Morocco (n = 2) or because they were considered to be secondary cases (n = 2). Two of the cases were matched with only one control respectively. Cases were more likely than controls to have eaten shrimps (mOR: 5.6; 95% CI: 1.1–28), to have resided on the Moroccan coast (mOR: 9.3; 95% CI: 1.1–78) and to have eaten shrimp in a restaurant (mOR: 11.1; 95% CI: 1.3–92.5). Cases were also more likely to have eaten in a restaurant before symptom onset (crude OR: 6.2; 95% CI: 1.1–295; we could not estimate the mOR because of the small number of cases). Consumption of squid was more frequent among cases (43%) compared with controls (15%), but the association was not significant (Table 2).

**Table 2:** Assessing associations between exposures and cases of *Salmonella* Chester infection by univariate conditional logistic regression, France, 2014 (n=14 cases)

Exposure	Cases (N=14)		Control-cases (N=26)		Matched OR^b^	95% CI
	n	%^a^	n	%^a^		
**Meat**						
Beef	12	92	20	80	2.7	0.26–28
Lamb/sheep meat	5	42	14	54	0.5	0.12–2.2
Chicken	11	79	22	85	0.6	0.12–3.2
Chicken sausage	3	21	4	16	1.3	0.22–8.0
Turkey ham	1	7	8	31	0.2	0.02–1.7
Cachir	2	14	6	23	0.6	0.10–3.5
Poultry meat sandwich	2	25	5	24	1	0.05–19
**Milk and eggs products**						
Pasteurised milk	6	55	16	76	0.2	0.02–1.7
Yogurt	8	57	19	76	0.4	0.06–2.2
Spreadable cheese	7	54	16	64	0.7	0.08–5.3
Scrambled eggs	6	49	6	29	3	0.54–16
**Vegetables and fruits**						
Tomato	11	78	18	69	1.4	0.33–6.0
Cucumber	9	69	16	64	1.2	0.28–5.3
Grapes	10	71	18	72	1	0.27–0.7
Melon	9	64	20	77	0.6	0.12–2.8
Water melon	10	71	21	81	0.5	0.08–2.7
Olives	7	50	15	60	0.7	0.16–3.1
**See food and fish**						
Sardine	6	43	8	31	1.5	0.44–5.0
Shrimp	7	50	4	15	5.6	**1.1–28**
Squid	6	43	4	15	3.3	0.81–14
**Sweets**						
Ice cream	10	71	15	60	1.6	0.36–7.2
Popcorn	4	31	8	32	0.9	0.21–0.9
**Eating place**						
Fast food X attendance	4	29	5	19	1.9	0.39–8.9
Restaurant attendance	14	100	18	69	6.2	1.1–295^c^
Shrimp consumption in restaurant	6	43	1	4	11.1	1.3–92.5^c^
**Place of residence**						
Residing on the coast	11	92	9	41	9.3	**1.1–78**

CI: confidence interval; OR: odds ratio.

^a^ Percentages are based on the number of cases or control-cases who answered the questionnaire about a given exposure. These numbers can be less than the totals provided in respective column headers.

^b^ OR matched on age categories and week of symptom onset.

^c^ Crude odds ratio.

### Microbiology

#### *Multidrug-resistant* Salmonella *Chester strains*

The AST showed that 63 *S.* Chester isolates (of 105 isolates tested, i.e. 60%) were resistant to at least nalidixic acid with a MIC range of 24–64 mg/L (Table 3 and data not shown). Among the 39 tested strains, which were acquired in Morocco (human and non-human, 2013–2015), 35 were resistant to at least nalidixic acid. Quinolone-resistant *S.* Chester isolates were also found from travellers returning from the African continent (Côte d’Ivoire, n = 1; Senegal, n = 1 or unspecified, n = 1) in 2014, from 13 French and English cases from 2014 and 2015 with no reported travel and from turkey meat imported from Spain in 2015. In silico MLST indicated that 61 of 63 quinolone-resistant isolates (including the 35 Moroccan ones) belonged to a new type, ST1954. This ST was also found in 15 quinolone-susceptible isolates (Table 3). The 61 quinolone-resistant *S.* Chester ST1954 isolates contained plasmid-mediated quinolone resistance (PMQR) genes. Seventeen isolates (28%) harboured a *qnrS1* gene associated with an IncN-pST7 plasmid (including the two Moroccan chicken sausage isolates) and 44 isolates (72%) contained a *qnrB19* gene associated with a Col plasmid (including the turkey meat, fishmeal and the sewage water isolates) (Table 3). Resistance to quinolones was only supported by these *qnr* genes, as no mutation was found in quinolone-resistant determining regions (*gyrA*, *gyrB*, *parC* and *parE* genes). Furthermore, a transposon belonging to the Tn*3*-like family was also identified and carried *strA*, *strB, sul2, tet(A*) and/or *floR* genes conferring resistance to streptomycin, sulfonamides, tetracycline, and/or chloramphenicol, respectively. The *floR* gene was only associated with the IncN-qnrS1 plasmid (Table 3).

**Table 3:** Microbiological characteristics of *Salmonella enterica* serotype Chester, European Union, 1937–2015 (n=153 isolates)

MLST	Number of isolates	Source (n)	Country of acquisition (n)^a^	Year (n)	AST profile^b^ (n)	PFGE type	Plasmid type_pMLST	Resistance genes patterns (n)
343	14	Human (14)	Reported none (4) Unknown (4) Cambodia (1) India (1) Indonesia (1) Maldives (1) Sri Lanka (1) Thailand (1)	2012 (1) 2013 (1) 2014 (3) 2015 (9)	Susceptible (4) Not tested (10)	XCHE_1887 (1) Not tested (13)	Absence (8) IncFII (6)	Absence (14)
411	23	Human (23)	Reported none (8) Unknown (8) Reported yes (3) Greece (2) Burkina Faso (1) Togo (1)	1937 (1) 2012 (2) 2013 (1) 2014 (13) 2015 (6)	Susceptible (15) ACroCazKGSuTmpTeNal (1) Not tested (7)	XCHE_1 (1) XCHE_3 (1) XCHE_4 (1) XCHE_1949 (1) Not tested (19)	Absence (15) Col (2) incFII (6)	Absence (22) *strA, strB, sul1, dfrA18, tet(D),qnrB4,bla*_DHA-1_ (1)
1954	96	Human (90) Non human (6)	Reported none (18) Unknown (22) African continent (2) Côte d’Ivoire (1) The Gambia (1) Morocco (46) Netherlands (1) Senegal (4) Spain (1)	2011 (1) 2013 (4) 2014 (59) 2015 (32)	Susceptible (13) AKNTGNal (1) ASSpSuTmpCTeNal (1) ASSuTmpCTeNal (2) Nal (24) SSuTmpCTeNal (13) SuTmpCTeNal (1) SuTmpNal (1) SuTmpTe (2) SuTmpTeNal (18) Not tested (20)	Lane4 (1) XCHE_1440 (16) XCHE_2 (1) XCHE_2010 (4) XCHE_2011 (1) XCHE_5 (1) XCHE_X1 (2) Not tested (70)	Absence (11) Col (64) IncN_ST7 (19) IncI1 (6) IncX1 (1)	Absence (15) *qnrB19* (26) *strA, strB, sul2, dfrA14, floR, tet(A), qnrS1* (16) *strA, strB, sul2, dfrA14, tet(A), qnrB19* (33) *strA, strB, sul2, dfrA14, tet(A)* (2) *qnrB19, bla*_TEM-1_ (1) *strA, strB, sul2, dfrA14, floR, tet(A), qnrS1, bla*_TEM_ (3)
1965	5	Human (5)	Reported none (2) Unknown (1) Ghana (1) Senegal (1)	2012 (1) 2014 (1) 2015 (3)	Susceptible (2) Not tested (3)	Not tested (5)	Absence (1) IncFII (4)	Absence (5)
2063	15	Human (1) ATCC_11997	Reported none (2) Unknown (7) India (2) Sri Lanka (2) Thailand (1) Vietnam (1)	Unknown (1) 2014 (6) 2015 (8)	Susceptible (5) SuTmp (1) ASuTmpNal (1) Not tested (8)	XCHE_1951 (1)	Absence (11) IncI1 (2) Col (2)	Absence (13) *strA, strB, sul2, dfrA14 (1)* *strA, strB, sul2, dfrA14, qnrS1, bla_TEM_* (1)

AST: antimicrobial susceptibility testing; MLST: multilocus sequence type; PFGE: pulsed-field gel electrophoresis.

^a^ For the country of acquisition ‘reported none’ indicates that the patients specified that they did not travel prior to the two weeks before their onset of symptoms, while ‘unknown’ indicates that no information was available as to the country of acquisition of the strain.

^b^ A: ampicillin; C: chloramphenicol; G: gentamicin; K: kanamycin; N: netilmicin; Nal: nalidixic acid; S: streptomycin; Sp: spectinomycin; Su: sulfonamide; T: tobramycin; Te: tetracycline; Tmp: trimethoprim.

#### Pulsed-field gel electrophoresis patterns

Two main pulsed-field gel electrophoresis (PFGE) patterns, XCHE_1440 and XCHE_2010, were observed among the outbreak isolates by using the ECDC cluster detection tools (data not shown). A few other patterns were also observed among the ST1954 outbreak strains.

#### Whole genome sequencing analysis

**Figure 2:**
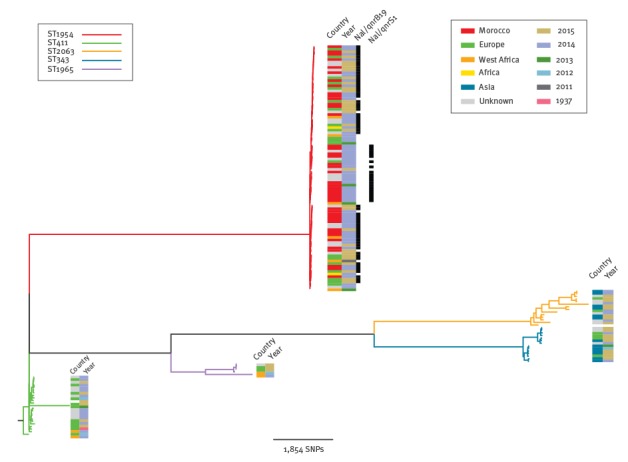
Phylogenetic tree of *Salmonella enterica* serotype Chester, European Union, 1937–2015 (n=153 isolates)

The WGS results showed that 153 human and non-human *S.* Chester isolates clustered phylogenetically into five tight groups. The grouping was concordant with MLST distribution, ST1954 (n = 96), ST411 (n = 23), ST2063 (n = 15), ST343 (n = 14) and ST1965 (n = 5) (Figure 2).

Nal: nalidixic acid; SNP: single nucleotide polymorphism; ST: sequence type.

Maximum-likelihood phylogenetic tree based on the analysis of 11,879 chromosomal SNPs from the 153 short read sequences of *Salmonella* Chester using harvest v1.0.1 f parsnp function against the 17K reference strain and rooted on the 201009678 genome. Branches distributing in a cluster corresponding to a given ST are assigned a specific colour and different colours are respectively used for ST1954, ST411, ST2063, ST343 and ST1965. The geographical area of acquisition/collection of the strain and the year of isolation are shown on the right of each branch together with information as to whether the strain has a Col/qnrB19 plasmid or an IncN/qnrS1 plasmid (whereby presence of either plasmid is indicated by a black box). The remaining quinolone-susceptible isolates are indicated by the absence of a black box (in particular for all the non-ST1954 isolates).

The SNP difference among the 96 isolates of the ST1954 cluster ranged between 0 and 214 SNPs; the ST1954 cluster itself was at a distance of 8, 453 SNPs from the reference strain.

**Figure 3:**
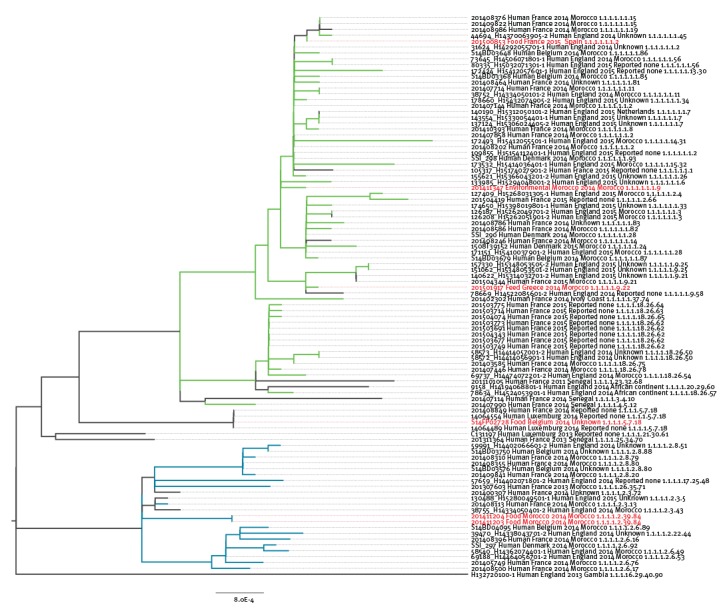
Maximum Likelihood phylogeny of the *Salmonella* Chester ST1954 strains, European Union (n=96 strains)

Within the ST1954 outbreak-cluster the SNP distance between strains was between 0 and 214 SNPs, the cluster itself being 8,453 SNPs distant from the reference 17K genome. The epidemic ST1954 clone encompassed all *S.* Chester QnrS1- and QnrB19 producers of the outbreak period as well as the 15 ST1954 quinolone-susceptible strains that had been isolated since 2011. Furthermore, all the six non-human *S.* Chester isolates were distributed throughout this cluster and some of them had < 5 SNP of difference with human cases (Figure 3).

ST1954 strains were mapped to the Spades v2.5.1 de novo assembly of isolate 60056_H14424061601–2 using BWA-MEM. The taxa are labelled with the strain number, isolate source, country of origin, year of isolation, travel information, and single nucleotide polymorphism (SNP) address. Hierarchical single linkage clustering was performed on the pairwise SNP difference between all isolates at various distance thresholds. The SNP address is a seven number indicating the range of SNPs as follow: Δ250, Δ100, Δ50, Δ25, Δ10, Δ5, Δ0. In green, the QnrB19-producing isolates; in blue, the QnrS1-producing isolates; in black the susceptible-quinolone isolates and in red, the non-human isolates.

## Discussion

This multinational outbreak of *S.* Chester cases associated with travel to Morocco has affected at least six EU countries since 2014. The true extent of the outbreak has probably been larger than observed, with unreported cases both in visitors to and residents of the affected area. Morocco is a popular holiday destination welcoming more than 10 million international travellers in 2014. The most common countries of origin of visitors registered at the Moroccan border were France (n = 3,494,112 visitors) and Spain (n = 2,134,610) [28]. In relation to the populations of the country of residence, the highest proportions of travellers to Morocco in 2014 came from France (5,405/100,000 inhabitants), Belgium (5,320/100,000 inhabitants) and Spain (4,567/100,000 inhabitants). This could explain the predominance of French residents among outbreak cases. The very young age of the cases we report could be an observational bias because we caught cases who had consulted a medical doctor after their return to Europe. These cases were more likely the very young and more severely affected by salmonellosis. 

In September 2014 and during the investigation, Moroccan health authorities were kept informed by ECDC and SpF and attended telephone meetings held to discuss the event. The French ministry of health informed in October 2014, through the International Health Regulation mechanism, the Moroccan ministry of health of the increase in *S.* Chester cases among French travellers returning from this country. Outbreaks of food-borne infection, only, are reportable in Morocco. No *S.* Chester outbreak was reported by the Moroccan authorities before and during the investigation. 

The epidemiological investigations suggest that the source of the outbreak was in Morocco. We found significant associations between *S.* Chester infection and shrimp consumption, visiting the coast and restaurant attendance before symptoms. The OR associated with squid consumption was high although it did not reach statistical significance. These results suggest that seafood, shrimp in particular, could be one of the sources of this outbreak. Multiple other sources of human contamination are suggested by the molecular and WGS analysis of the non-human strains: the chicken sausage could explain the human cases with isolates carrying the incN-*qnrS1* plasmid that appeared in 2014 and the turkey meat, some of human cases of 2015 with the Col-*qnrB19* positive isolates. The fishmeal and the decanted water samples, also contaminated by Col-*qnrB19* strains, may indicate the possible contamination of the environment by Moroccan poultries. Interestingly, fishmeal has been a major component of industrialised poultry feed [29]. Thus, to explain this contamination of different food chains, further environmental studies in Moroccan flocks are needed to highlight the potential cross-contamination/transmission mechanisms. The Col-*qnrB19* and incN-*qnrS1* types of plasmids have been widely described in *E. coli* and *Salmonella* from animals, the environment and humans worldwide [30] but IncN-pST7 plasmid has never been reported to date.

We compared the exposures of controls to the exposures of cases divided into two different groups according to the two plasmids’ distribution in their *S.* Chester strains. However, this analysis did not reinforce existing associations or highlight any new association between an exposure and the *S.* Chester infection (data not shown). Only 50% of cases could be explained by self-reported consumption of shrimp. This low proportion might be due to the recall period bias or to the fact that shrimp is a stealth food vehicle used in many common dishes (salads, pizza, sauce). No association between poultry (chicken and turkey) consumption and *S.* Chester infection could be identified, probably because chicken is widely consumed by the population (79% of the cases and 85% of the controls in our study) and due to the low power of our study. Poultry and seafood are very commonly implicated in *Salmonella*-related food-borne outbreaks [31,32]. Furthermore, seafood and chicken meat have been identified, along with beef, as products most involved in the spreading of *Salmonella* in Morocco [33]. *Salmonella* contamination of the Moroccan coast, between 2002 and 2005, has already been shown in previous studies [34,35]. Moreover, according to the RASFF database, various serotypes of *Salmonella* were found 27 times in fishmeal from Morocco during the period ranging from January 2010 to June 2015 (at least six *S.* Chester) [13].

The number of reported *S.* Chester cases in affected EU countries decreased after week 37 (mid-September) 2014 probably because most travellers came back from Morocco before the beginning of the school year. Indeed, we observed a new increase in number of *S.* Chester cases in September 2015 with 55 cases (at least 16 with travel history to Morocco) in France, 36 cases in Belgium, seven cases in Spain and four cases in Denmark. In 2016 in France we observed an increase again with 70 cases on the same period (between April and October), 16 had travel history to Morocco. Retrospectively, we also observed in France a slight increase in numbers of *S*. Chester cases during the summer 2013 with 14 cases during the period August–September 2013 compared with four in 2012 for the same period. The hypothesis that there are persistent sources of contamination in Morocco but also, to a lesser extent, in other West African countries is raised. In case of persistent sources and if no control measure is taken in Morocco a new increase could be observed every summers.

There were several limitations to our investigation. First, we observed that symptom onset of most of cases occurred at the end of their stay in Morocco or after they were back in France. The investigation probably missed cases who were sick during their stay and whose symptoms were already resolved before returning to France. Moreover, cases did not accurately represent all French tourists visiting Morocco, as only those seeking healthcare in France and that were tested for *Salmonella* were identified. Nevertheless, this should not affect the ORs, as the same limitations pertain to controls. We selected controls among other non-typhoidal *Salmonella* cases with other serotypes and matched them by age, travel to Morocco and exposure period. This could result in ‘overmatching’ and as a consequence lead to underestimating our ORs. One possible drawback of this design is that the aetiological exposures were different between serotypes, which could lead to false associations. Among our control group, nine different serotypes were included which would reduce this risk. The advantage of this design, in comparison with healthy people as controls, was the likely reduction of the recall bias, as ill people tend to recall different food exposures more accurately [36]. The case–case study design was already successfully used in several studies [37,38]. Unfortunately, we could not perform a multivariate analysis due to the small number of interviewed cases and controls. Finally, to our knowledge no environment and food investigations were conducted in Morocco.

The source(s) of this outbreak was located in Morocco, making it more difficult to investigate than an outbreak with a source in a EU country. However, the multinational collaboration was very helpful to share information for both the epidemiological and microbiological investigations. In this context, EPIS was very a useful tool. This kind of collaboration should be promoted and reinforced in case of outbreaks affecting several countries and occurring at a holiday destination. Specific recommendations for this outbreak were not taken because the risk posed by *Salmonella* in Morocco was already known and prevention and information messages already broadcasted.

In conclusion, this outbreak is probably a multi-source outbreak with several contaminated foods and likely also food chains. Chicken and shrimp in Morocco could be one of the sources of this outbreak. We recommend continuing collaboration and communication at EU level, in particular to report cases or new outbreaks through EPIS, and also to reinforce collaboration with Moroccan health authorities. Local epidemiologists could be involved in investigating such events in the field.
